# Analysis of evolution of the policy framework and governance mechanisms and their influence on the institutionalisation process of integrated community case management in Burkina Faso between 2010 and 2024: a scoping review

**DOI:** 10.3389/fpubh.2025.1672118

**Published:** 2026-01-26

**Authors:** Hamed Sidwaya Ouedraogo, Ahmed Kabore, Daouda Ouedraogo, Bètamou Coulibaly, Abdramane Bassiahi Soura, Maxime Koine Drabo

**Affiliations:** 1 Directorate General of Public Health, Ministry of Health, Ouagadougou, Burkina Faso; 2 Health Training and Research Unit/Health Sciences (UFR/SDS), Public Health Laboratory, Joseph Ki-Zerbo University, Ouagadougou, Burkina Faso; 3 Institute of Sports Sciences and Human Development, Joseph Ki-Zerbo University, Ouagadougou, Burkina Faso; 4 Higher Institute of Population Sciences, Joseph Ki-Zerbo University, Ouagadougou, Burkina Faso; 5 Institute for Health Sciences Research (IRSS)/CNRST, Ouagadougou, Burkina Faso

**Keywords:** Burkina Faso, childhood diseases, community dynamics, institutionalisation, integrated community care, policy ownership, stakeholders

## Abstract

**Context:**

Integrated Community Case Management (iCCM) has been implemented in Burkina Faso for several years. In view of the insufficient reduction in infant mortality due to multiple factors, strengthening its institutionalisation was recommended in 2019 after Addis Abeba conference. We conducted this study to understand how changes in the policy framework, governance of the system, and power relations between stakeholders have shaped the institutionalisation of iCCM from 2010 to 2024 in Burkina Faso.

**Methods:**

A documentary analysis using the READ approach (Review, Extract, Analyse, Distil) was used to select the documents. After identifying the relevant documents relating to the institutionalisation of iCCM in Burkina Faso (2010–2024) through double validation by two researchers, we used a grid to extract data, which was analysed to identify interactions between three key areas (policy framework development, governance and financing, exercise of power between actors and community involvement) with a view to institutionalisation in Burkina Faso.

**Results:**

Starting with a weak political framework in 2010, the pressures of political transitions (2015 and 2022), the ambitions of partners (combating diseases such as HIV/AIDS and malnutrition and achieving development goals), combined with pressures from the social, health and security crisis and research findings, ultimately strengthened political commitment to iCCM. The dominance of partners’ power linked to funding facilitated its integration into national health priorities, but limited its transformation into public health policy and its integration into the healthcare chain. Insufficient political commitment to iCCM and the absence of a rigorous regulatory framework for its funding limited the mobilisation of internal financial resources. These challenges were exacerbated by fragmented governance with low community involvement, which impacted implementation and limited ownership and local resolution of certain difficulties.

**Conclusion:**

The institutionalisation of iCCM in Burkina Faso has been partial. It has been hampered by partial commitment, a lack of a robust regulatory framework for its financing, fragmented governance and vertical management.

## 1 Introduction

As part of efforts to strengthen underserved populations in terms of healthcare (1), in 2012 the World Health Organisation (WHO) and the United Nations Children’s Fund (UNICEF) (2, 3) recommended that countries implement the Integrated Community Case Management (iCCM)as a complement to clinical care (4–6). This was intended to strengthen the availability of primary health care and the implementation of international guidelines (7, 8). Despite strong support from countries for its implementation (9, 10) and the progress it can bring (10), the Sustainable Development Goals (SDGs) in terms of infant mortality were far from being achieved (11). Mortality amongst children under five between 2010 and 2017 fell from 62 to 49 per 1,000 live births, which is still high compared to the SDG targets (12). During the analysis of the factors that limited the effects of this iCCM, gaps were identified in the integration of the strategy into national policies, a low level of funding that was very often fragmented (13), with numerous logistical problems hindering the availability of services (14). These difficulties had been identified in Burkina Faso since the 2011 community health assessment conduct as a national study [15]and persisted several years later with problems in financing inputs and capacity building for community-based health workers (CBHWs) (15–17), frequent disruptions and disparities in implementation (1, 9, 18). Strategic documents were developed to guide community health actions (19) and the profile of CHWs was formalised in 2014 (20). At the international level, the 2019 recommendation from the Addis Ababa conference on institutionalisation was intended to facilitate the mobilisation of health system actors to resolve these issues and ensure a decline in infant mortality. However, the fact remains that infant mortality remained high in 2023, estimated at 45 per 1,000 live births in Burkina Faso. Most of the various studies that have been conducted have focused on specific components of iCCM and have highlighted difficulties in strengthening the implementation of iCCM in several sub-Saharan African countries (21–24) despite the recommendation to institutionalise iCCM (14). The institutionalisation of iCCM was intended to ensure that it became an integral and sustainable part of the national health system through political, legal, financial and operational integration (14, 25). However, evaluations of institutionalisation, which have mostly been conducted in English-speaking African countries, have remained fragmented on issues such as institutional support and governance (26–28), or at times on factors affecting the quality of community health workers’ interventions (25, 29–32) or the implementation process and lessons learned (10, 21, 22, 33). A few studies that have focused on a comprehensive analysis of the institutionalisation of iCCM (34–36) have mainly emphasised components such as implementation, coverage analysis, logistics, or effectiveness, without addressing the underlying political and governance dynamics. These various conclusions did not enable us to understand why, after several years of implementation, iCCM has not yielded the expected results, with low using by households (1, 37) and infant mortality remaining high. In order to gain a better understanding of this situation, we initiated this study to examine how political developments, the governance of health systems, and power relations between the state, technical partners, and communities interacted to shape the institutionalisation of iCCM in Burkina Faso between 2010 and 2024. This research was based on a conceptual perspective of institutionalisation as described by Kok et al. and Oliphant et al. as a progressive, systemic change that reflects the political will and organisational commitment to permanently embed an initially exogenous strategy (iCCM in our case) into the endogenous norms and practises of the national health system (25, 38).

## 2 Methods

The methodology adopted was a documentary analysis based on the READ (Review, Extract, Analyse, Distil) approach proposed by Dalglish et al. (39). This methodology describes a systematic procedure for collecting and analysing documents produced or described in a health policy context at all levels (global, national, local) in four steps: (i) prepare the documents, (ii) extract the data, (iii) analyse the data, and (iv) synthesise the conclusions. We wanted to take advantage of this flexible approach, which facilitates the definition of document selection criteria and promotes the integration of relevant documents with practical advice at each stage of its application, incorporating epistemological and theoretical issues such as the socially constructed nature of documents and their role in modern bureaucracies (29). The use of the READ approach in describing its application methodology focuses on integrating various types of documents, facilitating robust triangulation. This was important for our study because it allowed us to avoid considering political documents as neutral sources of information, but rather as discursive references that embody institutional values, competing narratives and power configurations. It facilitates the understanding of the political dynamics and governance aspects that underpinned the institutionalisation of iCCM in Burkina Faso. Using this approach, we analysed the mechanisms of institutionalisation of iCCM in Burkina Faso, taking into account the interactions between the following thematic areas: (i) the evolution of the political framework and governance mechanisms, (ii) the exercise of power between stakeholders and its influence on the political framework and governance mechanisms, and (iii) the relationship with communities. This section presents the foundations of the analysis strategy, the criteria for including documents, the selection of relevant data and the analytical approach.

### 2.1 General analysis strategy

The READ approach adopted for document analysis in health policy research is a method that allows for the systematic examination of documents relating to public policies and strategies, particularly those produced by the government and its partners, evaluation reports, and related scientific publications. The READ analytical framework had the advantage of facilitating rigorous and systematic analysis of health policy documents whilst emphasising the context in which health policy emerged and was implemented, taking contextual factors into account. It facilitated a coherent and transparent synthesis of the document content.

To ensure the proper implementation of this READ approach, this work was based on a grid (Table 1) developed from theoretical frameworks already described for institutionalisation studies. The three frameworks that guided its development were (i) those developed by McGorman et al. which focused on describing the stages of introducing iCCM in countries following a linear process (40), (ii) the Addis Ababa reference framework, which defined nine components of iCCM institutionalisation based on McGorman’s reference framework, specifying the critical elements on which states should act to better integrate iCCM into the health system (14) and (iii) the framework developed by experts from the US President’s Malaria Initiative (PMI) (41), which defined the evolution of iCCM institutionalisation through to maturity but tended to define a gradual process that did not sufficiently integrate analysis of stakeholder power relations and community dynamics. This grid has better guided the extraction of relevant elements by capturing the chronological aspects of iCCM implementation as a health policy, the political trajectories necessary for understanding iCCM implementation in a country (40) and their implications for health, the identification of key actors and their interactions, the power dynamics between the state, partners and the community with the effect of diverging interests, alliances and negotiations (14, 24). The grid made it possible to avoid considering Iccm as a static element, to consider the different stages and to take into account the relevant aspects that interacted and shaped institutionalisation through the elements contained in the table (25, 27, 41).

**Table 1:** Elements of the model that guided the literature review and document analysis.

Areas of our model	Elements to be analysed (including interactions between areas)
1. Evolution of the policy framework and governance	National political events, local contexts (security climate, public health events), systemic change, political will adoption of legal texts, level of political commitment, clarity of roles, alignment between levels (central/local), role of PTFs, evaluation of cycles (implementation, maturation), existence of legal/regulatory frameworks for integrated community-based management of diseases (iCCM), integration into national policies and strategies, national community care development plan, alignment of interventions with national priorities, institutional effectiveness and implementation of strategies and operational planning, financing, organisational commitment and governance, coordination mechanism, coordination of strategy components, clarity of roles and responsibilities, degree of collaboration, monitoring/supervision
2. Distribution of powers amongst stakeholders (government, PTFs, communities) and resource allocation	Relationships between the government, NGOs, communities, technical and financial partners, negotiation dynamics and mechanisms for communication, coordination and collaboration, conflicts and synergies of interests, degree of influence, budget allocated to integrated management of childhood malaria, aid dependency, existence of specific budget lines, proportion of public funding, alignment with national priorities, facilitation of transfer/empowerment
3. Community involvement in implementation	Degree of community involvement in the process, consideration of community priorities, community ownership Functionality of mechanisms for community participation in health system management (health committees, various consultation frameworks at all levels of the system) level of communication between government structures, degree of social engagement and co-production of services, community financing, community control capacity and level of accountability of government actors to communities/community-based organisations, community representation

This harmonisation ensured consistency between empirical coding and the conceptual perspective of institutionalisation as a political process.

### 2.2 Inclusion criteria

Documents were selected according to the following criteria:

- They specifically addressed iCCM or one of the key elements of its implementation as defined by WHO and UNICEF (14, 42);
- They were produced by recognised institutions (government, international non-governmental organisations (NGOs), donors, research institutes);
- They contained relevant information on at least one of the following three elements: (i) policy developments, governance and financing of the strategy, (ii) interactions between actors and the exercise of power, (iii) interaction between the health system and communities;
- Were available in English or French;
- Were published between 2010 and 2024, to ensure that our analysis covered the period of the two national health development plans in Burkina Faso, including the 2011–2020 plan (19) and the current framework covering the period 2021–2030 (43). This range is also explained by the fact that the official recommendation to States for the application of iCCM at the international level was published in 2012 (2).

### 2.3 Document selection process

The selection process took place in three successive stages:

- Initial identification: physical collection of documents (strategic documents, programme and project reports) and consultation of institutional databases of the Ministry of Health of Burkina Faso, United Nations agencies such as WHO, UNICEF, other partners and cooperation agencies such as the US President’s Malaria Initiative (PMI), the Task Force on Child Health, as well as electronic scientific databases such as PubMed, Embase, CINHAL, SCOPUS, Web of Science and Google Scholar, based on a search strategy (Supplementary Table S3). This reading was carried out by HSO, OD, and CB.
- Sorting by relevance: we excluded documents that did not meet the inclusion criteria, content that did not address aspects related to the objectives of our study, PowerPoint presentations without analysis, short press releases without evaluative content, and duplicates. This sorting process was carried out by HSO, OD, and CB, and in cases of disagreement, we consulted AK, BAS, and MKD.

Thematic coding and data extraction: after selecting the documents of interest, we analysed them, extracted the relevant data, and then proceeded with coding. The themes were organised according to our three thematic areas of interest. We did this using conceptualised coding based on the themes of the three areas (Table 1), as is recommended when a predetermined framework and theme of interest are available (44), and the elements of interest were labelled in the documents. Two researchers (HSO and OD) carried out the coding independently, then compared and harmonised the categories to ensure reliability between coders. Triangulation was carried out by comparing data from different types of strategy documents (policies, evaluations, specific reports, guidelines, plans) and scientific publications, institutional perspectives (government, partners and civil society structures such as NGOs). This facilitated the analysis of developments in implementation according to the policy framework, governance dynamics, representations of interests and power relations. In addition, relevant articles selected for this documentary analysis and certain involved interviews with key actors, helped us to reduce interpretation bias and fill gaps inherent in the analysis of official documents, which sometimes fail to capture actual practises after decisions have been made. This cross-validation process strengthened the analytical robustness. In addition, reflective notes were used to document how the position and disciplinary orientation of researchers could influence interpretation. The analysis was conducted to identify convergences, divergences, gaps, and causal or dependency relationships between the components of the system.

### 2.4 Data analysis approach

The extracted data were analysed using thematic content analysis (44). The extracted data were recorded in an Excel spreadsheet (Supplementary File).

Using a deductive approach (45), we drew on the themes of the thematic areas described in Table 1 and conducted the analysis according to the three levels of health interventions implemented by the health system described by Zulu et al. (46). Although the literature review offers longitudinal and institutional depth, some limitations may remain, including the fact that these health policy and strategy documents reflect official discourse rather than informal practises, and that outputs produced within the framework of this iCCM are often influenced by donors. The diversity of the documentary resources analysed (evaluation reports and scientific articles) and this triangulation ensured rigour in the analysis of the results. The study also took into account all levels of the health system (from the community to the technocratic sphere) in the triangulation and contextual interpretation so as not to omit interactions that are very important in the study of health policies.

## 3 Results

**Figure 1:**
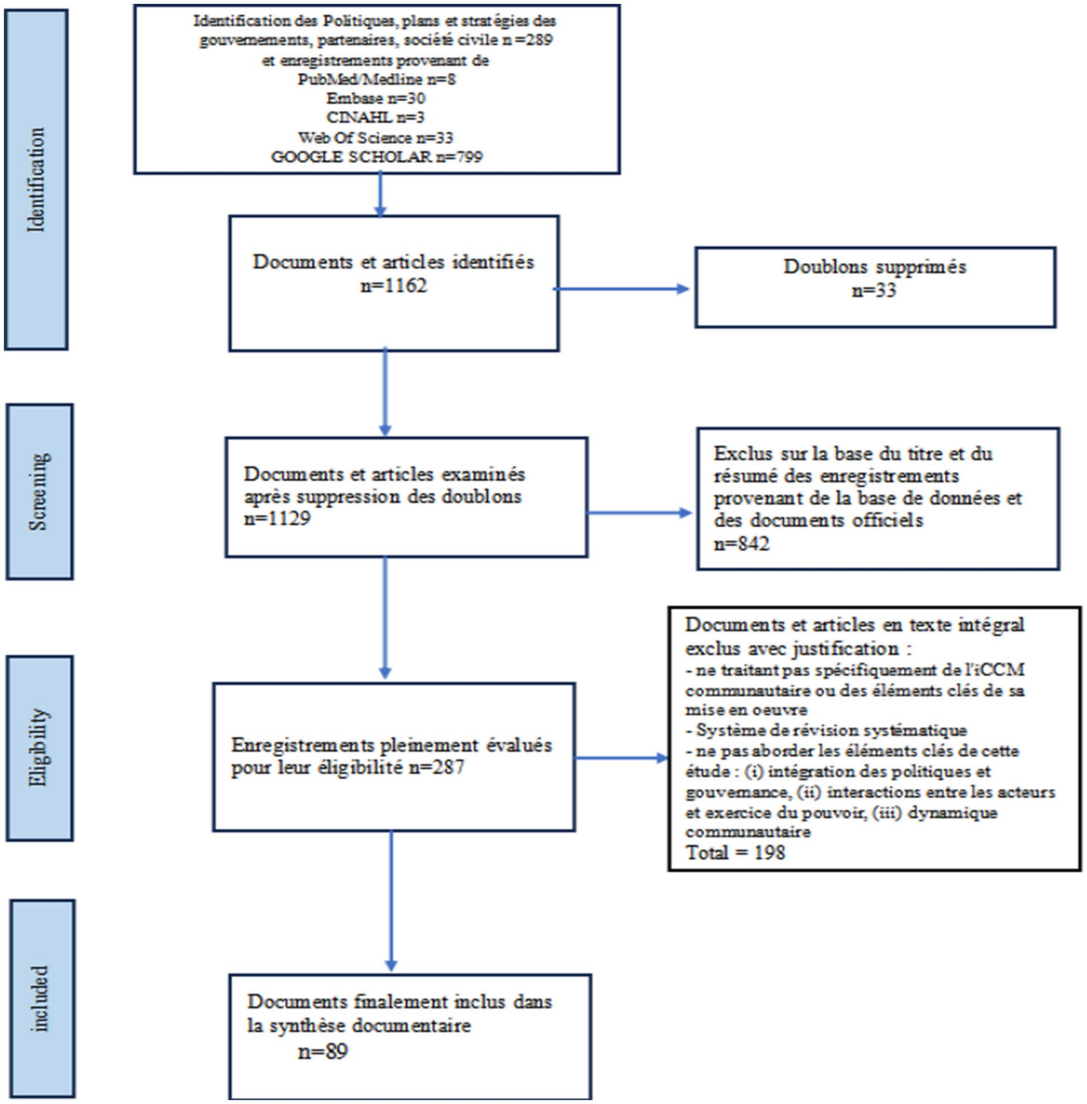
Diagram illustrating the selection process, grounds for exclusion and final number of documents.

A total of 89 documents were selected as part of the document selection process (Figure 1).

Analysis of these documents showed that the implementation of iCCM in Burkina Faso has been marked by successive political developments (see Table 2), changes in health governance and interactions between national, international and community actors. These interactions have been influenced by imbalances in decision-making power. Between 2010 and 2024, this process was shaped by several dynamics.

**Table 2:** Chronology of key events in the implementation of iCCM in Burkina Faso (2008–2024).

Phase	Period	Actions	Categorisation	References
Experimental phase and pilot implementation under the influence of technical and financial partners	2008	Pilot introduction of iCCM in a few districts (UNICEF, CHAI, PMI) Adoption of the first national community health strategy	Strategy testing phase and start of coordination of community interventions	(128), (9)
	2010	Development of the iCCM training manual adapted to Burkina Faso	Start of service standardisation	(129)
		iCCM mentioned in the 2011–2020 PNDS as a complementary strategy	Partial recognition of iCCM	(130)
	2014	Development of the CBHWs profile (roles, missions)	Structuring of community services and definition of the role of the community health worker	(131)
Leadership transition phase in implementation, expansion and improvement of geographical coverage	2016-2017	Adoption of CBHWs recruitment measures by the Council of Ministers Integration of iCCM into vertical policies (National Malaria Control Programme, SRMNEA)	Official and full recognition of ASBC, sectoral integration	(132), (133)
	2016	Drafting of the iCCM monitoring plan	Strengthening monitoring and evaluation	(132)
	2018	Extension of free healthcare to community health services	Strengthening the expansion of the strategy	(134)
Phase of strengthening stakeholder alignment in implementation	2019	Adoption of the National Community Health Strategy (SNSC) 2019–2023 recognising iCCM Revision of the ASBC profile	Strategic shift and strengthening of the role of CBHWs	(135)
	2019	Rollout of the health resilience strategy based largely on iCCM	Strengthening national ownership and adapting implementation	(135)
	2020	Integration of iCCM into the community investment portfolio and universal health coverage plans	Alignment with UHC	(136), (137)
	2021	PNDS 2021–2030: iCCM as a pillar of community access	Policy consolidation and maturation phase	(138)
	2022	Rollout of the transition, development and stabilisation plan		(139), (140)
	2023	Recruitment of volunteers/community health workers (National volunteers/CBHWs)	Phase covering peri-urban areas of major urban centres and areas with security issues	(141)
Maturation phase	2024	Adoption of the National Community Health Strategy (SNSC) 2024–2028 reaffirming iCCM	Programme consolidation	(142)

The period from 2010 to 2019 was generally marked by insufficient consideration of the strategy in the 2011–2020 National Health Development Plan (PNDS), with gaps in terms of programmatic and operational planning guidelines (47, 48). This void was also felt in the governance of implementation, which was marked by a duality between programs and central directorates rather than synergy. The result was governance dictated by technical and financial partners (choice of areas of intervention, actors to be involved at times, and styles of intervention) who were the financial arms of pilot projects according to their areas of intervention (1, 9, 15, 28). The majority of initiatives were driven by technical and financial partner(TFPs) (16, 19, 28, 31, 43, 49–51), with a vertical approach that was not well integrated into national mechanisms (9, 17, 24, 28, 52). Coordination was fairly limited and difficult (17, 49, 53, 54) due to the multipolarity that existed at the level of the central directorates of the Ministry of Health (17, 48, 55, 56). It was not until 2016 that the state began to regain control of the strategy’s governance, before consolidating it from 2019 onwards. This phase (2016–2019) corresponds to a period during which the country opted to strengthen health promotion as a strategic orientation in the PNDS (2011–2020). In 2014, the government decided to improve the quality of the profile of people working as community health workers (20) in response to pressure from partners to strengthen the community aspect of the fight against HIV/AIDS through contracts with community-based organisations that use community health workers (57, 58) on the one hand, and with a desire to correct the shortcomings identified by the community health assessment in relation to the diversity of community health worker profiles on the other (15). This highly political decision also aimed to meet the need for human capital development identified by the national development framework (SCADD) in response to the population’s difficult access to healthcare (47, 59).

The lack of policy focus between 2010 and 2014 (1, 9, 28, 60–62) has gradually given way to an attempt to integrate the strategy into its policies and programme documents from 2016 onwards (17, 48, 63), particularly those targeting children (63–67), in line with the ambitions outlined above. This evolution in the implementation of iCCM was truly driven by the political transition of 2015 (popular uprising at the end of 2014, the rise of military power in 2015 and election of a new president at the end of 2015) (68), which favoured the advent of a national economic and social development framework (69). The development of this framework was motivated by electoral considerations during the 2015 power transition from military to civilians. On the basis of this framework, the government established in 2016 took the decision to operationalise community-based health workers (CBHWs) (decision to recruit 16,000 paid $34 per month) (70). In addition, a policy of free healthcare for women and children was adopted and later extended to the community level in line with the social policy of the government at that time (71). The political adoption of iCCM was subsequently reflected in the priorities and planning guidelines from 2017 to the present (2025) (64, 72–74) and in operational planning, particularly the Ministry of Health’s consolidated action plans for 2021–2025 (75–77). In addition, the roles of CBHWs have been clarified and aligned with the revised set of care delivery guidelines, with the aim of strengthening consistency with the overall health policies of the PNDS 2021–2030 (43, 78). All these efforts by technical structures were aimed at operationalising the political decision to operationalise CBHWs and bring healthcare closer to communities (Figure 1).

The momentum was reinforced by the country’s participation in various international consultations that scrutinised the implementation of community-based interventions, including iCCM, with suggestions made to States and then incorporated into the Global Fund’s funding priorities (13, 14). Participation in these international consultations led to commitments to strengthen the institutionalisation of community health (79). The adoption of iCCM was also facilitated by the dissemination of scientific publications on the benefits of its full implementation and the shortcomings identified in pilot projects carried out mainly by partners (1, 15, 28, 52, 80–82). Finally, the security crisis that developed and worsened from 2019 onwards, as well as the advent of COVID-19, virtually dictated a transition in health policy towards the implementation of interventions aimed at strengthening the resilience of the health system (83), relying mainly on community actors to provide essential care packages (preventive and curative) (84–87). This has led to a stronger presence of donors in community health budgets, especially in areas of humanitarian intervention and iCCM. This combination of international pressure, factual data, internal political visions and the context of the security crisis has facilitated the launch of iCCM as a national priority and the beginning of the national financial resources mobilisation, largely devoted to paying CBHWs incentives (17, 88–92). The various policy decisions were taken without any real correction to the governance of the strategy, whose central coordination remained fragmented (50), thus failing to create the institutional conditions that would have given rise to the idea of developing a long-term vision for a well-integrated intervention in the health system This gap was reinforced by partners who strengthened their presence and provided targeted support to maintain control of the strategy (93). The direct consequence was insufficient funding, without explicit and specific consideration of a scheme to secure CBHWs inputs, whose procurement scheme was integrated into that of the health centres.

Despite political commitments and actions that provided strong institutional support for the strategy and guided the strengthening of iCCM implementation, it was hampered by insufficient funding, which remained very fragmented, external and dependent on the partners’ agenda until the advent of a new military and political transition in 2022. This new transition also established a new development framework that emphasised community health (94, 95) and laid the groundwork for a renewed strengthening of iCCM. The country adopted a new national strategic plan for community health (19) and recruited additional staff targeting urban areas and those facing security challenges in line with the need to strengthen the resilience of the health system (95). In addition, the multipolarity observed in coordination for more than a decade has been resolved by the establishment of a central directorate in charge of community health (96, 97) with full responsibility for coordinating all aspects of iCCM implementation. However, the level of funding has remained insufficient and predominantly external, limiting the leadership of the state (17, 92, 98). This funding from technical and financial partners exceeded 50% and was largely predominant for items such as the purchase of inputs, according to the Ministry of Health’s performance reports (91, 99).

The downside of this dynamic, which was largely driven by the partners who had been the main donors since the first phase in the 2010s (49, 100), is that the strategy was conceptualised by governments and communities as a project-based intervention and was ultimately not fundamentally integrated as a pillar of the system to be made fully functional with the necessary resources. This stagnation could also be explained by the absence of a multi-stakeholder regulatory framework (incorporating the financial arm of the government) (17, 48) to guide the consistent implementation of iCCM in Burkina Faso and ensure sufficient funding (48).

This has resulted in interruptions in the availability of services, creating disparities. In addition, the continuation of partner interventions targeting specific areas has accentuated these disparities in the availability of iCCM (17, 49, 50).

At the operational level, the inadequate implementation of guidelines for the management of free healthcare products in the community sector has not encouraged the use of state resources to supply inputs to CBHWs. This is because the system provided for the replenishment of CBHWs through orders for inputs from health centres, which was not effective (71). This scheme did not specify the potential benefits that would accrue to health teams, even though they received incentive bonuses calculated on the basis of the medicines purchased by the health centre. This situation therefore created a conflict of interest, leading to insidious and persistent malfunctioning. This failure of the supply system via health centres was therefore linked to a conflict of interest, as the full implementation of iCCM would lead to a reduction in the premiums paid to providers, part of which was indexed to the sale of medicines within the health centre and the number of treatments provided to children under 5 years of age (101).

Furthermore, the literature reviewed revealed persistent tensions related to overlapping responsibilities in the management of CBHWss, which were primarily responsible for implementing iCCM, which did not facilitate the planning and monitoring of interventions (17). Finally, certain inputs, such as dispersible amoxicillin, remained the responsibility of partners such as UNICEF for ordering and distribution in the field through this single channel, limiting the state’s capacity for action. The shortages reduced the use of CBHWs services, which were already unsatisfactory according to some authors (80). This low usage was therefore maintained by poor integration into the healthcare provision system, which remained centred on health centres run by healthcare providers, creating a social perception of unreliable services amongst communities with regard to iCCM (37, 80).

Faced with these shortcomings in operational institutionalisation, community representatives could have acted as a bulwark at times by challenging central governance and mobilising community resources. Unfortunately, however, they were only loosely involved in the process and did not participate meaningfully in decision-making (49, 102, 103).

The implementation of public health interventions calls for better coordination, and participatory monitoring and interaction mechanisms facilitate problem-solving and legitimisation by stakeholders (104, 105). However, in the case of iCCM implementation, attempts to establish coordination frameworks did exist at certain times. However, these frameworks have been largely ineffective due to insufficient funding, resulting in missed opportunities for accountability and alignment of interventions (19, 48, 103, 106).

Since 2019, strategic documents have placed greater emphasis on strengthening the interface between communities and health structures in order to improve their contributions (43, 78). However, community monitoring mechanisms for interventions implemented by the Ministry of Health did exist and demonstrated their ability to support interventions to combat tuberculosis, HIV/AIDS and malaria within the framework of financial support from the Global Fund (107, 108).

These results reveal a second power imbalance between Ministry of Health technicians and community representatives, limiting the decision-making power of communities in the process (61) and undermining their ability to advocate to government officials for endogenous financing of iCCM. Communities and their representatives are invited to participate in aspects related to the extension of formal services or at events that give the appearance of participatory governance, rather than as governance partners. Full implementation of the strategy requires balancing the balance of power and ensuring the full involvement of all stakeholders, including community organisations, at all stages.

The country has therefore implemented a iCCM with highly centralised and technocratic governance, which is poorly integrated into the healthcare system in health zones, constituting the first observation of isolated and/or rural populations, and which is not available to all communities on a continuous basis. iCCM in Burkina Faso is currently poorly institutionalised, especially at the operational level, due to a lack of institutionalisation of its funding, despite progress in its political and theoretical institutionalisation.

## 4 Discussion

The documentary analysis carried out showed that iCCM in Burkina Faso was gradually built up between 2010 and 2024 through a series of political reforms driven by high-level political choices, advocacy based on scientific evidence, and a desire to build a resilient health system to cope with the effects of socio-health and security crises, and a dominant relationship between partners due to financial power and influence over national priorities. It has been shaped by fluctuating governance, which has prevented the development of a highly integrated and autonomous system. Its implementation has also been weakened by insufficient interaction with communities. Its funding has remained predominantly external, jeopardising its sustainability.

The first phase of iCCM implementation was marked by weak political ownership, partner-dominated governance, and fragmented coordination. iCCM was insufficiently addressed in the 2011–2020 PNDS, as well as in strategic documents and programmes (45, 46). Its implementation was coordinated in a context of institutional duality between central directorates and vertical programmes, which led to limited and weak coordination (47, 83, 106, 107). This observed multipolarity complicated decision-making and communication with partners and weakened the state’s leadership (45, 47–49). The result was that TFPs played a dominant role in planning and geographical targeting through their influence on the prioritisation of interventions in the context of pilot project funding (1, 9, 15, 17, 26, 41, 83, 84). Interventions were therefore poorly integrated into national mechanisms (9, 22, 26, 47, 66) and heavily concentrated in certain areas of interest to partners, without taking into account the views of communities, which were considered only as beneficiaries. This reflects an extrinsic institutionalisation, dictated by the donors’ agenda rather than by a clear state strategy.

From 2016 onwards, the implementation of iCCM was increasingly part of an international dynamic in which interaction between governments and partners focused on the need to strengthen community health in national policies following assessments that the Millennium Development Goals had not been achieved and the new guidelines in the SDG roadmap (11). These international aspirations and the pressure exerted on the new political leadership in Burkina Faso (2016) to respond to the aspirations of the population in a context of insufficient human resources reinforced the development ambitions of iCCM through the full operationalisation of CBHWs (6, 109, 110). This gradual evolution in the political adoption of iCCM was in line with the stages described by McGorman et al. (40). However, the dynamic, which was somewhat biassed in an approach that was almost dictated by partners (100), did not allow for the development of an intervention that was strongly integrated into the health system with genuine ownership by the national side.

This trajectory for the introduction of iCCM in Burkina Faso was similar to that of other countries such as Nigeria (126) and Ethiopia (127), which went through the stages of formalising community health workers and budget planning, thus confirming a technical orientation with recommended phases following the steps of the McGorman model (40). This similarity in implementation trajectories, with a concordance of the stages described in international guidelines (6, 14, 40, 109, 111), strongly suggests an international agenda that has been dictated to developing countries through pressure exerted via funding priorities that effectively impose public health interventions on states (13, 38, 49).

In the case of Burkina Faso, the political transitions in both 2015 and 2022 have been beneficial for strengthening political adoption and implementation of innovation (especially the formalisation of the status of CBHWs by the state) and have advanced the operationalisation of iCCM. Allen et al. noted in their work the importance of political leadership and strategic competence at the state level for the success of community health reforms (27). Furthermore, the combined effects of the security crisis, COVID-19 and the international agenda to strengthen the institutionalisation of community health from 2019 onwards boosted this implementation dynamic and the decision to integrate iCCM into the health system (83, 86). However, these political commitments and contextual factors did not have the expected effects on the financing of the strategy (17, 50, 92). Their effects were limited by a lack of consolidated coordination between several central structures with joint responsibility, without any synergy of action to carry out the advocacy necessary to mobilise resources commensurate with the challenges of integrating iCCM into the system. Central governance lacked the relevant analysis to reverse the trend of predominantly external funding in favour of genuine integration into healthcare provision systems and continuity of care provision at the community level. This leads us to conclude that bureaucracy caused the system to miss opportunities to definitively resolve this funding issue in light of the possibilities for reform offered by these periods of political transition.

Furthermore, the regulatory framework failed to create a strong demand for funding from the Ministry of Finance, and iCCM therefore remained marginal in national budget lines, with a heavy dependence on external aid for the acquisition of inputs, especially (17, 50, 71, 112, 113). The absence of a legal text institutionalising community health with details on how to implement flagship interventions such as iCCM and strengthening governance has been a structural obstacle to the full integration of iCCM into the system (13, 14, 38). The country has also failed to plan for real empowerment due to the co-financing model that has been put in place (43, 93, 114), which has been maintained and reinforced by the various financial commitments made by donors in support of the government’s response to the humanitarian crisis.

The experience of Burkina Faso reflects a recurring problem in developing countries, where there is little alignment between political decisions and planning cycles (especially in ministries responsible for finance), resulting in underfunding of priority interventions (24, 38, 49, 54, 115). Similar situations have been reported in several countries, such as Senegal, the Democratic Republic of Congo (DRC) and Malawi (22, 116, 117), where political commitments have not been followed up with real funding for the strategy. Funding has therefore remained largely external and heavily dependent on TFP priorities (14), with an asymmetry of power that has limited national leadership and led to fragmentation in implementation. From the partners’ perspective, external funding can be seen as both a catalyst and a constraint. The influence of donors and international NGOs, whilst contributing greatly to the implementation of the strategy (111), has limited the full exercise of national sovereignty over strategic orientations (14, 34, 118).

As for the strategy integration process, it ran into differences of interest due to conflicting rationales between service providers, who needed to work to increase the number of patients using the health centre in order to receive performance and bonuses, CBHWs, who needed to work and reduce the number of patients using the health centre (101). Thus, the adoption of free healthcare, which was supposed to accompany the process by providing inputs, suffered from a lack of implementation of standard operating procedures. This shortcoming was linked to the of the divergent interests of healthcare actors (motivation bonuses from the sale of health products from the health centre and the care of patients referred to as “acts”) (101) but also to the irregularity of follow-up and supervision visits, which created a lack of monitoring, leading to the chronicity of operational problems. This has not ensured the effective availability of inputs at CBHWs (71). This lack of monitoring has also been exacerbated by the fragmentation of coordination and the duality reported between programmes, which has led to a failure to systematically share data on the operational implementation of the strategy. This lack of monitoring was also exacerbated by the absence or non-functionality of consultation frameworks at different levels, which effectively established technocratic governance. This left little room for the establishment of mutual accountability between state actors and community structures (8, 14, 119). When frameworks were put in place, they did not sufficiently involve communities, especially at the decentralised level, despite the mechanisms provided for in the various national community health strategies (17). The lack of accountability maintained a divide between health structures and communities, creating a lack of mutual recognition and a lack of strong community contribution to implementation. Participatory monitoring mechanisms have also been criticised for a lack of balanced communication with health teams in health centres, a lack of technical and financial capacity to influence local decisions in this management, and their roles are often perceived as consultative rather than deliberative (25, 102, 120). This has relegated community actors to implementation roles as mere healthcare assistants or social mobilisation actors, without any real power of co-decision (121, 122). These gaps in community participation undermine the confidence of the population and do not help to dispel the perception that the care provided by CBHWs is of inferior. Quality Similar situations have been highlighted by other studies that have denounced the top-down nature of health policies, particularly in several sub-Saharan African countries, leading to failures or unsustainable results (22, 119, 123). The experience of other countries such as Uganda and Tanzania has also demonstrated the need to formalise institutional links between communities and formal systems in order to avoid the marginalisation of local actors (124, 125). In concrete terms, the implementation of iCCM in Burkina Faso can be described as both a success and a mirror. Firstly, it is a success in that it has managed to engage political actors in its adoption and has begun its institutional integration, with gains in terms of expanding access to healthcare for certain populations. Secondly, it is a mirror reflecting the broader structural challenges of governance dependent on external financial support, with power asymmetries and issues affecting certain key actors in the implementation mechanism, limiting the operationalisation and decision-making capacities of certain stakeholders.

To sustain progress, not only are more resources needed, but also a rebalancing of relations between donors, the state and communities, all underpinned by a rigorous and demanding regulatory framework that prevents blockages by technocrats with divergent interests.

Like any study, ours has certain methodological limitations.

It is based exclusively on available documents, without interviews with stakeholders, even though some of the articles used were based on interviews with them. This may limit our understanding of informal dynamics. In addition, some documents (unpublished or internal) could not be included, which may limit the additional information provided. Finally, this study only took into account studies published in English and French, which could constitute a bias for this literature review.

## 5 Conclusion

At the end of this study, we can affirm that political developments have led to systemic changes with the introduction of CBHW as a provider of curative care at the community level and a gradual shift in the prioritisation of iCCM in strategies and operational plans, reflecting a political will to ensure effective operationalisation. This political commitment has remained partial, as it has not been able to reverse the dependence on external funding, which weakens national leadership. Furthermore, the institutionalisation of iCCM has been hampered by a flawed organisational structure that has been unable to meet the challenges of its structuring and integration. This lack of unified coordination has prevented the technical work from being carried out and the advocacy necessary for the production of legal instruments that could facilitate the allocation of sufficient budget lines to meet the implementation needs of iCCM. The exercise of power relations between the state and its partners has kept this strategy in its exogenous status without genuine integration into public policies. Operational difficulties are perpetuated by divergent interests that are not taken into account in the structuring of free community healthcare. All of these shortcomings have led to operational difficulties that limit integration into the healthcare system with effective continuity to achieve routine service provision.

The main contribution of this study is to highlight a double asymmetry of power: external symmetry between the state and TFPs on the one hand, and internal symmetry between formal structures and communities on the other. The external asymmetry has limited the effective completion of ownership with sufficient budgetary allocations for routine implementation, whilst the internal asymmetry has limited the co-construction of interventions. To remedy this, the completion of community health reform is more than necessary. To do so, the government could ensure that (i) the institutional and financial framework for iCCM is strengthened through a clear, specific legal framework that allows for stable financing and covers all the needs of CBHWs, capable of reducing external dependence and having an impact on health centre attendance (and, moreover, the cost of free healthcare), (ii) clarify the mandates of the central structures involved in its implementation and build unified coordination, (iii) better formalise and revitalise the frameworks for interaction at different levels (central, regional and district, taking into account village general assemblies).

## Data Availability

The original contributions presented in the study are included in the article/Supplementary material, further inquiries can be directed to the corresponding author.
