# Supplementary material for: Analysis of evolution of the policy framework and governance mechanisms and their influence on the institutionalisation process of integrated community case management in Burkina Faso between 2010 and 2024: a scoping review
Source: Front Public Health. 2026 Jan 26;13:1672118. doi: 10.3389/fpubh.2025.1672118 (PMC12883763; doi:10.3389/fpubh.2025.1672118)
Supplement: Supplementary file 1 [file Table_1.DOCX]

Supplementary Material

# Supplementary Data

Supplementary file with Data in Excel: EXTRACTION DATA FILE.xlsx

# Supplementary Tables

**Table S1: Types of documents that can be consulted in the context of health policy studies**

| Category | Examples of documents |
| --- | --- |
| Official documents | - Policies or policy guidelines - Sectoral strategies or strategies relating to specific health issues - Statistical surveys or publications - Online press kits |
| Implementation documents | - Training manuals or working tools (brochures, clinical files, etc.) - Mid-term/final reports or evaluations - Financial analyses or mapping - Operational plans |
| Legal documents | - Decree - Ordinances |
| Working documents | - Annual reports - Performance reports - PowerPoint presentations |
| Scientific work | - Scientific publications - Master's or doctoral theses - Textbooks and other course materials |
| Media and communications | - Online press articles - Blogs and web pages |

**Table S2: Questions for document analysis**

| *Categorization of appropriate periods for analysis* | Type of question |
| --- | --- |
| *When analyzing individual documents*: | - Is the document complete? Is it a finished document or a draft? - What is the purpose of the document? Who is its target audience? - Under what circumstances was the document produced? Under what circumstances is it consumed? - Who created the document? In addition to the authors cited, who else is likely to have contributed to its creation? - What might be the "agenda" of the document's creators? - Are there other versions of the document? Why? How might they differ? - Are there any internal contradictions in the document (e.g., different justifications or frames)? - Is the document credible? Do you have any questions about its accuracy, good faith, balance, selective reasoning, etc.? - What sources are cited (or not cited)? What type of evidence does it use? |
| *When analyzing all of the documents:* | - How complete is the set of documents? What is missing? - Which documents were easy to find? Which were more difficult to find? Which proved impossible to find? Why is this the case? - What voices are represented in the documents as a whole? Which ones are not? - How do the documents compare in terms of content? How do they compare in terms of style, format, length, and "look"? What about formality and tone? - What visual information can you find in the documents (tables and graphs, images, etc.)? - How are the same issues addressed differently from one document to another? - Do the documents "talk to each other"? Do they refer to each other or respond to each other's arguments or proposals? Do they respond to other documents not included in the analysis? - How are the documents similar or different across domains, document types, or levels of governance (e.g., global, national, subnational)? - How does the information drawn from the documents compare with data from other sources (e.g., interviews, focus groups, observations, quantitative analyses)? |

**Table S 3: Research strategies**

| *Electronic database* | Strategies developed |
| --- | --- |
| *PubMed* | ("community-based management of childhood illness"[tw] OR "iCCM"[tw] OR "Malaria"[Mesh] OR "Diarrhea"[Mesh] OR "Pneumonia"[Mesh] OR )  AND ("Community Health Workers"[Mesh] OR "community health worker"[tw] OR "CHW"[tw] OR  "CBHW"[tw] OR "ASC"[tw] OR "ASBC"[tw] OR "community health services"[tw] OR "community-based care"[tw])  AND  ("Program development"[Mesh] OR "Health plan implementation"[Mesh] OR "implementation science"[tw] OR "program implementation"[tw] OR "scaling up"[tw] OR "institutionalization"[tw] OR "implementation"[tw])  AND  ("Burkina Faso"[Mesh] ) |
| EMBASE | ("community management of childhood illnesses":ti,ab,kw OR "iCCM":ti,ab,kw OR  'malaria'/exp OR 'diarrhea'/exp OR 'pneumonia'/exp )  AND  ("community health worker"/exp OR "community health worker":ti,ab,kw OR "CHW":ti,ab,kw OR  'CBHW':ti,ab,kw OR 'ASC':ti,ab,kw OR 'ASBC':ti,ab,kw OR 'community health services':ti,ab,kw OR  community care':ti,ab,kw )  AND  ("program development"/exp OR "health care planning"/exp OR "implementation science":ti,ab,kw OR "program implementation":ti,ab,kw OR "scaling up":ti,ab,kw OR "institutionalization":ti,ab,kw OR  institutionalization':ti,ab,kw OR 'implementation':ti,ab,kw )  AND  ("Burkina Faso"/exp OR "Burkina Faso":ti,ab,kw) |
| CINHAL | ("community-based management of childhood illnesses" OR "iCCM" OR (MH "Malaria") OR  (MH "Diarrhea") OR (MH "Pneumonia") )  AND  ((HM "Community health workers") OR "community health worker" OR CHW OR "community health services" OR "community health services" OR "community health services" OR "community health services"))  ASC OR "community health services" OR "community-based care")  AND  ( (MH "Burkina Faso") OR "Burkina Faso") |
| SCIENCE NETWORK | TS=("community management of childhood illnesses" OR "iCCM" OR "malaria" OR  "diarrhea" OR "diarrhea" OR "pneumonia")  AND  TS=("community health worker" OR "CHW" OR "CBHW" OR "ASC" OR "ASBC" OR  "community health services" OR "community-based care" )  AND  TS="Burkina Faso") |
| SCOPUS | ( TITLE-ABS-KEY("community management of childhood illnesses") OR  TITLE-ABS-KEY("iCCM") OR TITLE-ABS-KEY("malaria") OR TITLE-ABS-KEY("diarrhea") OR  TITLE-ABS-KEY("diarrhea") OR TITLE-ABS-KEY("pneumonia") OR TITLE-ABS-KEY("program development") OR TITLE-ABS-KEY("program implementation") OR  TITLE-ABS-KEY("institutionalization") OR TITLE-ABS-KEY("institutionalization") OR  TITLE-ABS-KEY("implementation"))  AND  ( TITLE-ABS-KEY("community health worker") OR TITLE-ABS-KEY("CHW") OR  TITLE-ABS-KEY("CBHW") OR TITLE-ABS-KEY("ASC") OR TITLE-ABS-KEY("ASBC") OR  TITLE-ABS-KEY("community health services") OR TITLE-ABS-KEY("community-based care"))  AND  ( TITLE-ABS-KEY("Burkina Faso")) |
